# The Three Genetics (Nuclear DNA, Mitochondrial DNA, and Gut Microbiome) of Longevity in Humans Considered as Metaorganisms

**DOI:** 10.1155/2014/560340

**Published:** 2014-04-24

**Authors:** Paolo Garagnani, Chiara Pirazzini, Cristina Giuliani, Marco Candela, Patrizia Brigidi, Federica Sevini, Donata Luiselli, Maria Giulia Bacalini, Stefano Salvioli, Miriam Capri, Daniela Monti, Daniela Mari, Sebastiano Collino, Massimo Delledonne, Patrick Descombes, Claudio Franceschi

**Affiliations:** ^1^Department of Experimental, Diagnostic and Specialty Medicine Experimental Pathology, University of Bologna, Via S. Giacomo 12, 40126 Bologna, Italy; ^2^Interdepartmental Centre “L. Galvani” (CIG), University of Bologna, Piazza di Porta S. Donato 1, 40126 Bologna, Italy; ^3^Center for Applied Biomedical Research, St. Orsola-Malpighi University Hospital, 40138 Bologna, Italy; ^4^Department of Biological, Geological and Environmental Sciences (BiGEA), Laboratory of Molecular Anthropology & Centre for Genome Biology, University of Bologna, Via Selmi 3, 40126 Bologna, Italy; ^5^Department of Pharmacy and Biotechnology, University of Bologna, Via Belmeloro 6, 40126 Bologna, Italy; ^6^Department of Clinical, Experimental and Biomedical Sciences, University of Florence, Viale Morgagni 50, 50134 Florence, Italy; ^7^Department of Medical Sciences, University of Milan, 20122 Milan, Italy; ^8^Geriatric Unit, IRCCS Ca' Grande Foundation, Maggiore Policlinico Hospital, 20122 Milan, Italy; ^9^Nestlé Institute of Health Sciences SA, Campus EPFL, Molecular Biomarkers Core, Quartier de l'Innovation, 1015 Lausanne, Switzerland; ^10^Department of Biotechnologies, University of Verona, Strada le Grazie 15, 37134 Verona, Italy; ^11^Nestlé Institute of Health Sciences SA, Campus EPFL, Functional Genomics Core, Quartier de l'Innovation, 1015 Lausanne, Switzerland; ^12^IRCCS, Institute of Neurological Sciences of Bologna, Ospedale Bellaria, Via Altura 3, 40139 Bologna, Italy; ^13^CNR, Institute of Organic Synthesis and Photoreactivity (ISOF), Via P. Gobetti 101, 40129 Bologna, Italy

## Abstract

Usually the genetics of human longevity is restricted to the nuclear genome (nDNA). However it is well known that the nDNA interacts with a physically and functionally separated genome, the mitochondrial DNA (mtDNA) that, even if limited in length and number of genes encoded, plays a major role in the ageing process. The complex interplay between nDNA/mtDNA and the environment is most likely involved in phenomena such as ageing and longevity. To this scenario we have to add another level of complexity represented by the microbiota, that is, the whole set of bacteria present in the different part of our body with their whole set of genes. In particular, several studies investigated the role of gut microbiota (GM) modifications in ageing and longevity and an age-related GM signature was found. In this view, human being must be considered as “metaorganism” and a more holistic approach is necessary to grasp the complex dynamics of the interaction between the environment and nDNA-mtDNA-GM of the host during ageing. In this review, the relationship between the three genetics and human longevity is addressed to point out that a comprehensive view will allow the researchers to properly address the complex interactions that occur during human lifespan.

## 1. Introduction

Longevity is a complex trait whose genetics has been extensively studied since many years. Understanding the genetic makeup that predisposes to longevity is an urgent challenge owing to the explosion of the elder population in western as well as in emerging countries.

Usually the studies on the genetics of human longevity are restricted to the analysis of nuclear genome (nDNA). However, another essential genome, that is, the mitochondrial genome (mtDNA), is part of the genetic machinery of each cell. Despite its limited length, the mtDNA encodes for few genes that constitute a quantitatively relevant group because of the high copy number of mtDNA in each cell.

These two genomes do not work in the void and life/survival, as well as ageing and longevity, depends on their complex interaction with environment/lifestyle. To this scenario we have to add another level of genetic complexity represented by the microbiota, that is, the whole set of bacteria that live in different anatomical districts of our body with their whole set of genes (microbiome). Indeed, the most comprehensive view is to consider human being as a “metaorganism” resulting from the close relationship with symbiont microbial ecosystems. A particular attention has been recently devoted to the gut microbiome (GM). The GM probably represents the most adaptable genetic counterpart of the human metaorganisms, being extremely plastic in response to age-related physiological changes in diet and modification in lifestyle.

Thus, the result of the ageing process is defined by the sum of a number of factors both biological and nonbiological (environmental and stochastic). Therefore while the ageing research based on the study of animal models starts assuming the existence of major genes that determine longevity, in humans this assumption represents an oversimplification. The study of human model imposes a more holistic view of the genetics to grasp the complex dynamics of the interaction between the environment, stochasticity, and the three genetics of the host (nDNA, mtDNA, and GM).

The main aim of this review is to sum up the state-of-the-art of the knowledge of the three genetic components in human longevity to demonstrate that within this comprehensive view the genetics moves from a crystallized concept (genes are forever) to a much more flexible and dynamic perspective, in which the complex interaction between genetic makeup and environment across the long-lasting human lifespan is properly addressed.

## 2. The Nuclear Genome

The study of genetics of ageing in human being is tangled given the high complexity of the interaction between heterogeneous environmental factors and genetic variability across a long period of time. A strategy for disentangling this complexity is to focus on robust human models of longevity such as centenarians.

Centenarians are a model of successful ageing as in most cases they display medical histories free from most of the major age-related diseases, including cancer, dementia, diabetes, and cardiovascular diseases. Their ability to reach the extreme limit of human life—escaping, or largely postponing, age-associated pathologies—is the result of the combination of a well-preserved and efficient immune system, a good capability to cope with different stressors, an appropriate lifestyle, and a robust genetic background [1–5].

At the beginning, studies on longevity were conducted on lower organisms and animal models, providing evidences that longevity could be influenced by many conserved genetic variants with small effects [6]. Complying with this idea, several association studies have been conducted, comparing centenarians' genetic profile to that of younger cohorts. Several gene variants have been found to be associated with longevity, including* IL6* -174 C/G [7],* IL10* -1082 A/G [8, 9],* PON1* gene [10],* SOD2* 401nt T/C [11], the arginine to proline amino acid substitution in* TP53* gene at codon 72 [12–15], and insulin/IGF1 signal response pathway [16–21], but replication studies have provided contradictory results [22].

This can be due to different reasons that include the effect of population structure [23] and the lack of an appropriate control group [24]. The best control group for centenarians should include subjects born in the same birth cohort since younger subjects could be grown up in very different environmental and socioeconomic conditions. Furthermore, the recruitment of an elderly cohort until the age of 100 is demanding also from the “experimental” point of view. Considering that in Italy 1/4000 individuals is centenarian, to perform a study with 100 centenarians researchers should consider a pool of about 400000 people and a much larger cohort to perform a longitudinal study with a final cohort of a hundred of survivors over 100 years of age [25]. This calculation clearly explains the difficulties in carrying out longitudinal studies on human longevity that include the extreme decades of life.

Technological advances in the last 10 years have fostered the study of the genetics of complex traits by means of genome-wide approaches that allow the simultaneous analysis of thousands of genetic variants on large cohorts.

Many genome-wide association studies (GWASs) have been conducted assuming that long-lived individuals could share several common genetic variants that influence human lifespan.

Nebel et al. genotyped 1848 Germans, 763 individuals aged 94–110 years and 1085 controls aged 45–77 years, and replicated the results on an independent cohort of 1614 subjects. They reported a statistical significant association only for one SNP, rs4420638, that is located near* APOC1* and is in linkage with* APOE* [26].

Deelen et al. [27] compared 403 unrelated nonagenarians and 1670 younger controls from the Leiden Longevity Study cohort and tested the emerged SNPs on 4149 nonagenarians and 7582 controls from the Rotterdam Study, Leiden 85-plus study, and Danish 1905 cohort. Only rs2075650 is associated with survival to old age also in the replication stage (OR = 0.71, 95%CI = 0.65–0.77, and *P* = 3.39∗10^−17^). This SNP is located in* TOMM40* gene, close to* APOE* gene and, even if it displays only a moderate linkage with* APOE *ε*4* determining variant, authors report an* APOE* dependent effect of rs2075650 on longevity.

Another GWAS was performed by Sebastiani et al. [28] considering initially 801 long-lived individuals (95–119 yrs) and 914 matched controls from the New England Centenarian Study. They identified 281 SNPs (about 130 genes) that they used to build a genetic risk model to distinguish cases (long-lived) from controls with 89% sensitivity/specificity. It is interesting to note that about 50% of the SNPs included in the model are located in intergenic regions, underlining that the regulatory machine plays a major role in longevity. This model has been tested in two independent cohorts (253 centenarians + 341 controls and 60 centenarians + 2863 controls) providing results that are less exciting than those obtained from the training datasets (60–58% and 78–61% sensitivity/specificity, resp.). Overfitting problems are common when high-dimensional data are managed and may play a role also in the relatively poor accuracy reported for the proposed model on the test datasets. Furthermore, authors reported that sensitivity of the predictor increases with increasing age, supporting the hypothesis that the influence of genetics on longevity gets stronger with increasing age. Moreover, this approach showed that different genetic signatures can be used to group centenarians into different “longevity classes” according to factors such as the prevalence or the age of onset of age-related diseases [29]. Nevertheless, some centenarians did not show a genetic signature of exceptional longevity, suggesting that exceptional longevity might be better explained by rare or private genetic variants. The 281 SNPs include rs2075650 in* TOMM40* gene but the contribution of this variant to the predictive power of the genetic risk model is poor. This is probably because of the low frequency of GG genotype that is almost absent in centenarians (frequency: 0.1%) and that makes the prediction of lifespan of AG and AA carriers uncertain without further genetic information [28]. It is relevant to note that this particular SNP (rs2075650) shows a cline in minor allele frequency from south to north Europe and this stresses the importance of stratifying models by ancestry.

Sebastiani and colleagues tested the 281 identified SNPs on 5 studies of centenarians from USA, Europe, and Japan and they found that 128 SNPs reached statistical significance, bringing out biological pathways deeply involved in exceptional longevity [30].

*APOE* is the only gene accounted as a “longevity determinant” by several independent GWASs.* APOE*ε** variants have been extensively analyzed and the frequency of **ε*4* allele has been found decreased in long-lived subjects [31–33] but this result varies among different populations [34, 35]. Recently, Tan et al. proposed a method to identify the signature of mortality deceleration at late age. They estimated the effect of* APOE*ε*4* variant in the Danish 1905 Birth Cohort and they found that relative risk of **ε*4* allele does not increase linearly with age, supporting the idea that this allele exerts its deleterious effect also in the last decades of life [36].

Overall, GWASs have proved to be rather disappointing for identifying genetic determinants of complex traits, that is, longevity or many age-associated diseases, and established loci account only for a small proportion of trait heritability. The lack of robust results can be attributed to different reasons that include, among others, the need to conduct these studies on hardly available large cohorts, the phenotypic heterogeneity of longevity, the gene-environment interactions, and the failure to identify both low frequency variants with large effects and rare variants. One point to take into account is that each cohort in GWASs could be characterized by population-specific genetic makeup. This phenomenon has been suggested by De Benedictis and Franceschi [37] from the observation that the demographic males/females ratio among centenarians is consistently different depending on the geographic origin of individuals. The authors sustain that this observation provides evidence of the existence of a genetics characteristic of each population and it makes less and less impressive the results of GWASs, which include different populations, even more difficult to interpret. The advent of next-generation sequencing (NGS) has renewed interest and hopes of the researchers in the study of the genetics of longevity, as this technology allows a previously unattainable systematic discovery of low frequency variants in thousands of samples. In the context of longevity, NGS has been applied to assess whether ageing is accompanied by an accelerated accumulation of somatic DNA mutations that affects the primary structure of proteins, ultimately compromising organismal functions [38]. Ye et al. performed whole genome sequencing of two pairs of monozygotic twins aged 40 and 100 years old, by using two independent NGS platforms and validating potentially discordant single-base substitutions by Sanger, Roche 454, and Ion Torrent sequencing. Authors found that NGS can detect somatic single nucleotide substitutions and that getting centenarian is not accompanied by an increase in the number of detectable somatic mutations. Furthermore, the authors highlighted that the low background somatic variation reported within twin pairs is advantageous if discordant twins are considered for the identification of disease-related mutations.*‬‬*

Studies carried out so far were unable to discriminate between two basic assumptions, that is, whether the ability to reach the extreme limits of human life is due to the presence of polymorphisms in “longevity genes” that promotes the achievement of old age or to the absence of harmful variants [39]. The study of extreme phenotypes has proved particularly useful to clarify these clinical issues [40]. In the field of longevity, this approach should include centenarians and individuals severely affected by age-associated diseases. Recently, interesting results were reported using this method to study type 2 diabetes (T2D). 1349 Italians, including 562 T2D patients, 558 unrelated controls, and 229 centenarians, were genotyped for 31 SNPs mapping within or nearby genes involved in T2D. rs7903146 in* TCF7L2* gene showed a progressive increase in the frequency of risk genotype (TT) from centenarians to diabetic patients who developed one or more complications and the strongest genotypic association was detected when diabetic patients were compared to centenarians (*P* = 9.066∗10^−7^). The use of centenarians in this kind of studies has proved to be useful to evaluate the biological relevance of genetic variants emerging from GWASs. Authors speculate that if a SNP is considered a statistically significant but “weak” risk factor for the disease and it is present at similar frequency in centenarians and in patients, its biological relevance can be assumed as negligible. On the contrary, if the frequency of a genetic risk variant is much higher in patients than in centenarians, it is likely that this SNP plays a consistent biological role in the pathogenesis of the disease under study [41]. It is also interesting to note that the T risk allele and the TT risk genotype were present in few centenarians, suggesting that they were not able to foster the T2D phenotype, likely because of being counteracted by healthy lifestyle.

In addition to centenarians, a widely used model to investigate longevity consists of long-lived families. Indeed, it was observed that siblings of centenarians have an increased relative risk of reaching 100 years, sustaining the existence of a heritable component of human longevity [42–44]. This heritable component has been estimated to account for about 25–32% of the observed variation in human population, with an increasing influence after 60 years of age [42, 45, 46]. To understand whether this heritable component results mostly from the genetics or from the familial environment, Schoenmaker et al. [47] proposed to consider spouses of long-lived subjects as an additional control group. They included in their analysis 100 families with at least two long-living siblings and they evaluated standardised mortality ratios, finding a survival benefit for all siblings of the long-living participants, for their parents, and for their offspring but not for their spouses. This result allowed authors to sustain that the families considered are genetically enriched for extreme survival and that it is unlikely that environmental factors play a major causative role in familial longevity. The environment effect on longevity was studied also by Montesanto et al. [48] on 202 long-lived families from Calabria. Authors compared the survival functions of nonagenarians' siblings to those of their spouses to estimate the genetic component of longevity, minimizing the effects of environmental factors. Authors confirmed that both parents and siblings of the nonagenarians had a significant survival benefit. They also reported for the first time a gender-effect restricted to males. Indeed, only male siblings showed a substantial survival advantage and the presence of a male nonagenarian in a family significantly decreased the mortality rate throughout lifetime for all the siblings, suggesting that genetic factors in males strongly affect the possibility of becoming long-lived. Family studies of exceptional longevity were performed also to identify rare genetic variants that cannot be discovered with population-based studies. One of the largest European projects aimed at identifying genes involved in healthy ageing, the Genetics of Healthy Ageing (GEHA) project, was focused on a sophisticated familial model of longevity, that is, nonagenarian sib pairs, that is, two or more siblings aged 90 years or older [49, 50]. During GEHA project, 2535 families comprising 5319 nonagenarian siblings were recruited from 15 regions in 11 European countries. In addition, younger persons aged 50–75 years were included as unrelated controls but coming from the same geographical area as the sib pairs. The comprehensive phenotype description and an estimation of the survival rate of a subset of GEHA subjects were performed on this exceptional cohort to identify survival predictors [51]. In particular, some predictors of longevity, including sociodemographic, physiological, clinical, and haematochemical parameters, were examined at baseline and 6 years after the recruitment in 1160 Italian GEHA 90+ siblings. It was observed that better specific cognitive and functional parameters (SMMSE, ADL scale, and hand grip strength), self-reported health, and clinical parameters (haemoglobin, creatinine, and total cholesterol) in 90+ sib-ships were also effective survival predictors. Cevenini et al. also suggest that this combination of the parameters identified in the GEHA model of healthy ageing is influenced by familiarity/genetics [51].

A genome-wide linkage analysis on 2118 European nonagenarian full sibships of the GEHA project was performed to identify chromosomal regions involved in longevity [52]. By using Illumina HumanLinkage12 Genotyping BeadChip, four regions (14q11.2, 17q12-q22, 19p13.3-p13.11, and 19q13.11-q13.32) were identified, together with three loci that were linked to longevity in a sex-specific manner (8p11.21-q13.1 (men), 15q12-q14 (women), and 19q13.33-q13.41 (women)). A GWAS performed in the same GEHA 90+ sibships and controls showed that only rs4420638 was significantly associated with longevity. As expected by results obtained in other genome-wide studies, this SNP tags the linkage disequilibrium block harboring the* TOMM40*,* APOE*, and* APOC1* genes. The analysis of* APOE*ε** variants in GEHA nonagenarians siblings showed that**ε*4* allele frequency was significantly lower than that reported for the geographically matched younger controls (6.8% versus 12.7%). In agreement with this finding, **ε*4* allele carriers have about 50% lower chance to become nonagenarians than the non-**ε*4* carriers (OR = 0.48, 95%CI = 0.42–0.55).

## 3. The Mitochondrial Genome

Several theories on ageing process and longevity posed mitochondria in a central position. Mitochondria produce the cellular energy through oxidative phosphorylation (OXPHOS) and many metabolic pathways are located in these organelles as well as the pathway controlling apoptosis. The two main mechanisms that link mitochondria to ageing are the mutagenesis of mitochondrial DNA (mtDNA) and the production of reactive oxygen species (ROS). The relative contribution of these two mechanisms and their interplay in the ageing process are still matter of debate, but a detailed analysis of the history of these theories is out of the scope of this review and it is well described elsewhere [53]. However, the more recent hypothesis states that ROS generation is not* per se* a cause of ageing, but rather a consequence of the age-dependent accumulation of mtDNA damage. Indeed, it is well known that mtDNA mutations increase with age, and recent findings show that this increment is likely due to errors in replication machinery or to unrepaired damage, placing ROS mutagenic effect in the background. Two studies support this hypothesis: (i) a study by Kennedy et al. [54] showed that in mice the mtDNA damage during ageing is not characterized by an accumulation of transvertions (i.e., the type of mutations caused by ROS) but by a higher prevalence of transition; (ii) the study of Trifunovic et al. [55] showed that defects in the proofreading function of gamma polymerase (pol*γ*) lead to a high accumulation of mtDNA mutations and to a consequent premature ageing of mice that carry these defects.

### 3.1. Longevity and mtDNA Somatic and Inherited Variability

Recent GWASs on age-related diseases [56–59] have identified some nuclear loci implicated in mitochondrial bioenergetics (*PPAR*γ*, PGC-1*α*,* and* UCPs*) providing further support to the hypothesis that mitochondria play a central role in the ageing process [60–62]. In humans, the processes influenced by mitochondrial activity and their effects on degenerative diseases and ageing appear to be modulated by mtDNA common variants as addressed by many studies [60, 63–73].

Recently, the association between recurrent or sporadic mtDNA mutations and longevity has been highlighted by the results of the GEHA project. In this project the control region of 3000 samples and the complete mtDNA of 1292 samples were sequenced including 650 ultranonagenarians (90+) and a comparable number of controls, enrolled in Denmark, Finland, Southern Italy, and Greece [74]. A haplogroup classification and a specific analysis for evaluating the burden of nonsynonymous mutations in different mtDNA regions were performed [75]. The results showed that the number of nonsynonymous mutations in mtDNA genes coding for subunits of OXPHOS Complexes I, III, and V is different between 90+ subjects and controls. In particular, the presence of mutations on complex I may be beneficial for longevity, while the cooccurrence of mutations on both complexes I and III or on both I and V might be detrimental to attain longevity. As haplogroup *J* is characterized by mutations in complex I genes, this result might explain previous contrasting findings emerging from association studies on *J* haplogroup and longevity [65, 67, 70, 76–78]. This result points out the need of complete sequencing of mtDNA in this type of genetic studies and the inadequacy of studies based on haplogroup classification. Intriguingly, the analysis of mtDNA sequences in 90+ and controls has also shown that many mtDNA mutations associated with a variety of mitochondrial and degenerative diseases are as frequent in 90+ as in younger controls (e.g., 4336T > C mutation in the tRNAGln in Alzheimer's disease), supporting the idea that the effect of mtDNA mutations is highly influenced by the individual-specific genetic background (the combination of nuclear and mitochondrial genome variants), as well as by stochastic events [68, 79, 80]. In conclusion, a major result of the GEHA study is that the interaction between mutations concomitantly occurring on different mtDNA genes can affect human longevity [74]. Moreover, such an effect of mtDNA variability on longevity seems to be mainly due to the cooccurrence of rare, private mutations, which are not detected by haplogroup analysis.

The complex relationship between mitochondrial genetics and longevity has been further puzzled when somatic mtDNA variability is considered. Indeed in one cell many mitochondrial genomes exist and the cooccurrence of mutated and wild type copies of mtDNAs is named heteroplasmy. Many studies [81–83] showed an accumulation of heteroplasmy during ageing in different tissues (such as muscle, brain, etc.). Recent findings demonstrated that even low-frequency heteroplasmic mutations can be inherited from the mother [84] suggesting a potential role of these variants as primer to potentiate the effect of somatic mutations that accumulate during ageing [85]. This component of mtDNA variability is difficult to study, as heteroplasmy pattern observed in adulthood is a mixture of both inherited and somatic (acquired) mtDNA mutations. Moreover, the proportion of mutated mtDNA can vary according to the tissues and cells considered. Only few studies were able to establish a link between heteroplasmy and healthy ageing and longevity [86, 87] and even less studies addressed the role that these accumulations may have in promoting longevity. A high incidence of the C150T transition in centenarians' leukocytes was observed and a remodeling event of mtDNA replication origin associated with this mutation was hypothesized. These studies also indicate that the level of heteroplasmy at position 150 is similar between relatives and correlates in parent-offspring pairs, thus suggesting a genetic influence. However previous technologies (such as DHPLC, pyrosequencing) have not allowed a high-resolution analysis of mtDNA variants occurring at a very low frequency. The advent of NGS technologies, with their capability for very high coverage, allows the analysis of mtDNA mutations at very low frequency with high accuracy [88]. This new technology applied to powerful models of longevity, such as centenarians and their offsprings, is expected to answer some of the open questions in this hot topic.

## 4. Gut Microbiome

In humans most of the microorganisms reside in the intestinal tract and their role is so central that all these microbes are considered as an additional organ, characterized by its own genome [89]. The gut microbial community is predominantly bacterial and the most characterized part inhabits the distal colon where two bacterial phyla—the Firmicutes and the Bacteroidetes—constitute more than 90% of the total community [90]. The relationship between human GM and the host is highly plastic, with the potential to readily adapt to changes in diet, life style, and geography, as well as to the different host ages, defining a process which is fundamental to maintain host health and homeostasis [91]. This plasticity has been recently highlighted by David and colleagues [92] that reported the effects on GM composition of different diets, that is, one based on animal products and another one based on plant products. Authors observed that the short-term consumption of these two kinds of diet alters microbial community structure and bypasses the interindividual differences in the microbial gene expression.

The composition of the microbiota strongly impacts on the host health. Indeed, several studies report that the dysbiosis of the microbiome occurs in different chronic conditions, including obesity, inflammatory bowel diseases, and diabetes [93–96].

Regardless of whether it is a cause or a consequence of diseases, the GM can actively contribute to diseases consolidation. Indeed, several studies on disorders show that a pathologic phenotype can be transmitted from a diseased animal to a healthy recipient through the graft of the microbiota and this also applies to complex diseases where host genetics and environmental factors play a role [97, 98]. The first study that linked microbiota to human ageing dates back to 1908 when Metchnikoff postulated that ageing can be caused by gut microbiota dysbiosis [99].

The age-related changes in gut microbiota are very controversial and results from recent studies are not always concordant. In fact the study by Biagi et al. [100], performed on Italian subjects, showed that* Bacteroidetes* proportion remains unchanged in elderly, whereas in a study on Irish elderly [101]* Bacteroidetes* strikingly increase and overcome the* Firmicutes*. The influence of different cultural habits and lifestyle in the two populations considered is likely the major force underlying the observed differences [102], even if a “study effect” cannot be excluded [103]. This observation is obviously complicated by a high level of interindividual variability in the composition of gut microbiota. However, studies agree that old individuals are characterized by a lower GM diversity [100] and by an enrichment in “pathobionts,” that is, proinflammatory bacteria that usually are present at low concentration in healthy individuals [104, 105]. Interestingly, in centenarians this GM profile has been associated with inflammaging, a condition that is characterized by a high level of blood inflammatory markers. A shift in microbial composition towards an age-related pattern was observed in inflammatory disorders [106], supporting the proinflammatory nature of an aged-type microbiota. However, the causes and the effects of a direct association between microbiota modifications and immunosenescence and inflammaging are still unclear [107]. Recently, an alteration of GM functional profile was also observed in extreme ageing. The age-related trajectory of the human gut microbiome was shown to be characterized by loss of genes for short chain fatty acid production—well-known anti-inflammatory GM metabolites [108]—and an overall decrease in the saccharolytic potential, while proteolytic functions were more abundant in centenarians'GM than in the intestinal metagenome of younger adults. Treatments that include prebiotics/probiotics seem to produce beneficial effects on healthy ageing but an extensive review of these concepts is outside the purpose of this review and they are extensively described elsewhere [107].

## 5. The Remodeling Theory of Ageing

**Figure 1:**
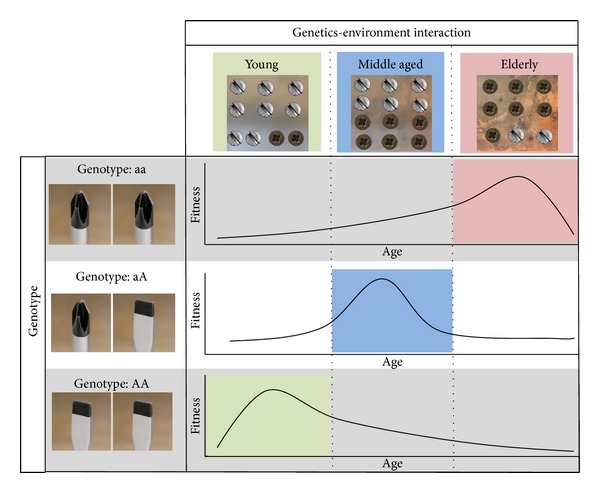
Schematic representation of the remodeling theory. The genetics-environment interactions as a function of age are represented as the fitness between the screwdrivers (genotype) and screw head shape (cross or cut). In particular, the different gene-environment interaction is represented by different ratio between the screw head shape. According to the remodeling theory, the interaction between the same genotype and time-related physiological conditions could result in different fitness, as shown in the three plots (fitness versus age). In particular, the highest fitness values for each genotype are coloured (red, blue, and green).

Human physiology undergoes profound changes from birth to old age. In the elderly, these changes are mainly the result of adaptive strategies at the molecular and cellular levels to compensate the damage accumulation that occurs over time. A major contribution to these changes is played by the cell microenvironment. A growing body of evidence supports the idea that the systemic environment is the repository of danger signal products and of the whole garbage that the senescent cells and the impaired tissues produce. Several studies provide evidence of rejuvenation of aged cells by exposure to a young systemic environment, suggesting that it is the microenvironment to which the cells are exposed to that causes/maintains the old phenotype [109–112]. Therefore, the assumption that a given allele has the same biological effect(s) in the body (systemic body environment) of young, adult, old, and very old people is simplistic. Within this perspective, the remodeling theory was postulated. This theory poses that the same allele has different effects on the probability of survival according to age-related physiological conditions (Figure 1), by modifying gene expression and, as a consequence, the composition of cell microenvironment which in turn modifies again gene expression in an amplifying vicious circle, which eventually is responsible for the systemic age-related decline. Indeed, as described above, the individual gene-environment interaction changes with age and a significant contribution to the remodeling could be provided by the three genomes interactions.

## 6. Mitochondrial-Nuclear Crosstalk

More than 90% of the factors required for mitochondrial function are encoded by the nuclear genome. Coevolution of mtDNA and nDNA is a crucial process that preserves biological functionality and cell activities [113]. This is demonstrated by Kenyon and Moraes who mark the interaction between nDNA and mtDNA, focusing on species-specific compatibility between these genomes [114]. In particular, using xenomitochondrial cybrids they showed that only our closest relatives, that is, chimpanzee and gorilla, were able to restore oxidative phosphorylation when placed in a human nuclear background, whereas distant relatives with a mtDNA that is more different from an evolutionary point of view, that is, orangutan and lemur, were not. It is also relevant to note that the time that leads to incompatibility between nuclear and mitochondrial genomes could be shorter than the divergence of species. Indeed, alterations in this equilibrium could alter cell functionality, leading to an increased disease susceptibility or pathological processes [115]. mtDNA is fundamental for energy production in the cell, and both nuclear and mitochondrial genes are needed to assemble the mitochondrial translation machinery and to carry out major processes, such as OXPHOS.

Similar evidences come from studies on* Drosophila* showing that epistatic interactions can cause incompatibilities that decrease fitness [116]. In particular, in* Drosophila,* the cooccurrence of naturally occurring mutations in a mitochondrial tRNA and in a nuclear-encoded tRNA synthetase that show little effect on their own severely compromises development and reproduction. The effect of this interaction affects mitochondrial functionality, supporting the hypothesis that the variable penetrance of mitochondrial DNA mutation is likely affected by mitochondrial-nuclear interactions [116].

### 6.1. From the Nucleus to the Mitochondria and Back

Interactions between nDNA and mtDNA products are possible at many levels such as protein-protein interactions in the OXPHOS, protein-RNA interactions in the mitochondrial ribosome, or nuclear factors-mtDNA recognition sites interactions in transcription and replication processes [115, 117, 118]. The interaction between these two genomes is bidirectional, meaning that there are both a flow of information from the nucleus toward the mitochondria and a mitochondrial retrograde signaling pathway.

Regarding the first type of communication (from the nucleus to the mitochondria), the nDNA encodes for all potential factors that regulate mtDNA replication, transcription, and processing, including mtDNA polymerase (pol*γ*). Nevertheless, nDNA encodes not only regulatory factors, but also structural proteins that constitute the respiratory multimeric protein complexes, as well as some noncoding RNAs that are imported subsequently into the mitochondria. Complex I contains 7 mtDNA gene products and at least 25 nDNA gene products, complex II contains no mtDNA gene products and 4 nDNA gene products, complex III is made of 1 mtDNA gene product and 10 nDNA gene products, complex IV contains 3 mtDNA gene products and 10 nDNA gene products, and complex V contains 2 mtDNA gene products and 11 nDNA gene products [119]. Regarding the nucleus-encoded RNAs, many import mechanisms exist but are still not fully understood [120].

Communication from the mitochondria to the nucleus involves metabolic signals (including ROS) but this field of research suffers from the lack of human studies. However, recent studies in* C. elegans* demonstrated that neuronal cells that experienced mitochondrial stress (i.e., electron transport chain (ETC) impairment) produce a signal that is transmitted from the mitochondria to the nucleus. Then, these cells produce other extracellular signals (called “mitokines”) that are able to induce a stress response (mitochondrial unfolded protein response) in the intestine, without altering the ETC functionality in intestinal cells. As it was observed that this mechanism extends lifespan, the authors speculate that a diffusible molecule released from one tissue might spread a sort of “longevity signal” to other tissues [121, 122].

The mechanisms that regulate the communication between these two genomes are not completely elucidated. To date, it is well known that the balance of the crosstalk between the nDNA and the mtDNA is essential for cellular homeostasis and events that perturb this delicate equilibrium increase the vulnerability of the cell and, thereby, the rate of ageing. Regarding the ageing process, a particular attention should be devoted to SNPs located in nDNA or in mtDNA that may affect the communication between these genomes. An interesting papers by Bertolin et al. [123] focuses on the role of TOMM machinery (a multiprotein complex responsible for importing most of mitochondrial proteins) in the mitochondrial clearance. Under normal conditions, damaged mitochondria are removed via autophagy but during ageing autophagy declines [124, 125], leading to accumulation of dysfunctional mitochondria. Bertolin and colleagues demonstrated that the degradation of the core structure of the TOMM complex is crucial in mitochondrial clearance and that mutations in* PARK2*, a cytosolic E3 ubiquitin-protein ligase recruited for proteasomal-mediated degradation of outer mitochondrial membrane, significantly affect the interaction with TOMM70A and TOMM40. These results let us hypothesize that also SNPs located in genes of the TOMM machinery could be involved in the effectiveness of autophagic mitochondrial clearance. From this point of view, rs2075650 in* TOMM40* that is associated to longevity, as previously described, could affect the relative configuration of the partners of TOMM complex, resulting in a more efficient clearance of damaged mitochondria. In this context, further studies are needed to elucidate the interactions between mtDNA and nuclear gene variants assessing their association with longevity in human.

The study of epistatic interactions represents a challenge [126] that is gradually becoming more achievable with the availability of NGS techniques. Indeed, the significant drop in costs and time commitment required to obtain a complete mtDNA sequence caused a burst in the number of available samples in both private and public databases [127]. It is noteworthy that about 22% of the whole database of the complete human mtDNA sequences was deposited in the last 12 months. The nDNA and mtDNA interactions could represent a critical issue when considered in the context of age-related heteroplasmy accumulations. Indeed, locally heteroplasmic mutations that alter this complex interaction can spread with age and impair cellular and tissues homeostasis.

## 7. Gut Microbiota-Host Genes Crosstalk and Role of the Diet

The three-way interaction between human genetics, environment, and microbiota fundamentally shaped the biological history of modern human populations and continues to affect healthy globally. The disruption of this interaction and stability by modifying one or more of these three components may be a trigger for the development of diseases.

A recent paper tried to determine to what extent the gut microbiota is determined by the host or by environmental factors, such as diet. This study demonstrated that the composition of gut microbiota among great ape species is phylogenetically conserved and pattern of relationship inferred from the analysis of the microbial communities was very similar to that inferred from host mitochondrial DNA, suggesting that host genome is a fundamental factor in determining microbial composition [128]. Ley and colleagues [129] suggested that both diet and phylogeny have driven the coevolution of mammalians and their gut microbiome. While, at large taxonomic scale, “diet” appears to be the major driving factor, at lower taxonomic scale, “phylogeny” well reflects microbiome composition. However, in certain circumstances, a highly specialized diet can override the phylogenetic inertia, resulting in an adaptive microbiome convergence of phylogenetically distant hosts that share a well-defined dietary specialization [130].

The role of human genetics in the assembly of the human intestinal microbiota is still controversial. Turnbaugh et al. [93] reported that monozygotic and dizygotic twins showed a comparable degree of similarity between their intestinal microbial communities. Since family members had a more similar intestinal microbiota profile than unrelated individuals, the authors concluded that the host genetics is secondary to environmental exposures in shaping the gut microbial ecology. However, another fingerprinting study of monozygotic and dizygotic twins showed a slightly reduced microbiota similarity profile in dizygotic twins [131].

A recent study in mice investigated how environmental stimuli and host genetic factors combine together to shape microbiota composition. In particular, this is the first study that tries to estimate the effects of maternal environment and host genetics. The results showed 18 host quantitative trait loci (QTL) in linkage with specific microbial taxa. In particular some of these loci affect species composition, others affect taxa, and others exert a pleiotropic effect. The authors suggest that the core measurable microbiota can be used in GWASs in humans [132].

Moreover, there is a clear evidence that host-microbe crosstalk involves the immune system, and in particular the gut-associated lymphoid tissue (GALT) likely developed to oversee the interaction between bacteria that live close to the intestinal mucosal surfaces [133]. In this process, enterocytes exert a very crucial role steering GALT toward tolerance or responsiveness, depending on the perceived degree of treats [134].

From an evolutionary perspective, the GALT-GM immunological crosstalk and the related tolerance of the immune system to the gut microbes have been a basal trait in mammalian evolution. Thus, the coevolutionary trajectories between mammals and their GM have been shaped by adaptive changes in the host genes that play a primary role in the crosstalk with the harbored microbial communities.

GWASs identified genes of both innate and adaptive immunity as relevant for inflammatory diseases, and interestingly several of these genes have been shown to have a role in shaping the gut microbial community. Genetic variants of several nuclear genes for components of immune system showed to have an impact on the composition of the gut microbiome. Mediterranean Fever (*MEFV*) gene encodes a protein called pyrin/marenostrin that controls inflammation by interacting with the cytoskeleton in white blood cells and that, when mutated, leads to an autoinflammatory disorder called “familial Mediterranean fever” [135]. Khachatryan et al. [136] demonstrated that mutations in* MEFV* gene are associated with a shift in the* Bacteroidetes*,* Firmicutes,* and Proteobacteria phyla and that patients with familial Mediterranean fever have a lower microbial diversity.* MYD88* gene is involved in communications between sensors of microbial products during inflammatory responses and loss of* MYD88* compromises the innate immune response to pathogens [137]. The microbiota from* MYD88*-deficient and wild-type mice was compared and it was found that three bacterial families (Lactobacillaceae, Rikenellaceae, and Porphyromonadaceae) differ in their relative abundance [138]. Studies on IgA locus were performed on both mice and humans. It was reported that mice deficient in IgA harbor an increase abundance of* Candidatus Savagella* [139]; furthermore, a decrease in proportion of IgA-coated bacteria in humans was associated with weight loss [140]. Other genes having roles in metabolism have been identified. APOA1 is the major component of high density lipoprotein in plasma and SNPs in* APOA1* gene have been associated with the risk of cardiovascular diseases [141] and with T2D [142]. Zhang et al. observed that the microbiota of* APOA1*-deficient mice has a different community structure in comparison with that of wild-type mice [143].

However, despite this clear coevolution between gut microbiota and host genomes, external stimuli are likely to play a crucial role in determining interindividual variability [144]. Diet is one of the external stimulus able to influence microbial compositions, and its effect on the microbiota is already reviewed elsewhere [145]. It has been demonstrated that a change from low in fat and rich in polysaccharide diet to a western diet, high in saturated and unsaturated fats, alters the microbial profile [146] and a high fat diet changes drastically the ratio between Bacteroidetes and Firmicutes. Furthermore, a recent study explores the intestinal microbiota from European children (characterized by western diet) and from children born in an African rural village in Burkina Faso (characterized by a fiber rich diet). The results suggest that consumption of sugar, animal fat, and calorie-dense foods in industrialized countries is rapidly limiting the adaptive potential of the microbiota by reducing functionality itself [147]. However, little is known regarding the relationship between environment and nuclear genetics in shaping this biodiversity. Studies by Franceschi and by other research groups clearly show that the ecology of the gut microbiota changes with age [100, 101, 104–108]. However, it is yet unclear how the drivers of this change combine with inflammaging, diet/environment, and genetic background. In any case it can be predicted that the age-related changes in the GM can stress and amplify the different genetic makeup of the host.

## 8. Conclusions: The Omic Challenge

This overview clearly shows that the knowledge of the genetics of human ageing relies on three pillars, that is, nuclear genetics, mitochondria genetics, and microbiome genetics. Until now this research field has suffered the fact that these three genetic domains have been studied separately, and it is urgent to set up investigations capable of analyzing these three genomics altogether. If these considerations are correct, the phenotypic characterization of the people/models used in genetic studies of ageing and longevity becomes critical, particularly regarding the lack of information of GM in most of the studies. The poor knowledge of GM genetics is critical because it is closely related to anthropological variables such as nutrition and diet. Similar considerations must be applied to the fact that mitochondrial genetics is not considered in GWASs, as the mitochondrial genome is closely related to human population evolution that is in turn molded by environmental adaptation to climate and diet. Finally, all the three genetic components of longevity change with age, and this is particularly evident in GM and mtDNA but it also applies to more subtle, but quantitatively more important, mutations in nDNA. Overall, the emerging scenario is that of a continuous and dynamic interaction between the different components of the genetics of human longevity that was unpredictable until recently and is currently largely unexplored.
